# Exposure to *Leptospira* spp. and Associated Risk Factors in the Human, Cattle and Dog Populations in Bhutan

**DOI:** 10.3390/pathogens10030308

**Published:** 2021-03-06

**Authors:** Anou Dreyfus, Marie-Thérèse Ruf, Anne Mayer-Scholl, Theresa Zitzl, Nadine Loosli, Nadja Seyhan Bier, Stephanie Hiereth, Sebastian Ulrich, Sven Poppert, Reinhard K. Straubinger, John Stenos, Tshokey Tshokey

**Affiliations:** 1 Department of Medicine, Swiss Tropical and Public Health Institute, 4055 Basel, Switzerland; therese.ruf@swisstph.ch (M.-T.R.); nadine.loosli@swisstph.ch (N.L.); sven.poppert@swisstph.ch (S.P.); 2 Medical Faculty, University of Basel, 4055 Basel, Switzerland; 3 Epidemiology and Clinical Research Unit, Institut Pasteur Madagascar, Antananarivo 101, Madagascar; 4 Department of Biological Safety, German Federal Institute for Risk Assessment, 10589 Berlin, Germany; anne.mayer-scholl@bfr.bund.de (A.M.-S.); nadja.bier@bfr.bund.de (N.S.B.); 5 Institute of Hygiene and Infectious Diseases of Animals, Justus Liebig University, 35392 Giessen, Germany; theresa.zitzl@t-online.de; 6 Chair for Bacteriology and Mycology, Institute for Infectious Diseases and Zoonoses, Faculty of Veterinary Medicine, Ludwig-Maximilians University Munich, 80539 Munich, Germany; s.hiereth@lmu.de (S.H.); ulrich@micro.vetmed.uni-muenchen.de (S.U.); or Reinhard.Straubinger@micro.vetmed.uni-muenchen.de (R.K.S.); 7 Australian Rickettsial Reference Laboratory, University Hospital Geelong, Geelong, VIC 3220, Australia; JOHN.STENOS@barwonhealth.org.au; 8 Department of Pathology and Laboratory Medicine, Jigme Dorji Wangchuck National Referral Hospital, Thimphu 11001, Bhutan; doc_tshokey@yahoo.com; 9 Faculty of Postgraduate Medicine, Khesar Gyalpo University of Medical Sciences of Bhutan, Thimphu 11001, Bhutan

**Keywords:** leptospirosis, microscopic agglutination test (MAT), seroprevalence, cattle, yak, dog, one health, Bhutan

## Abstract

Leptospirosis is a neglected worldwide zoonotic bacterial disease with a high prevalence in subtropical and tropical countries. The prevalence of *Leptospira* spp. in humans, cattle and dogs is unknown in Bhutan. Therefore, we sought to find out whether humans, cattle or dogs had been infected in the past with leptospires by measuring antibodies in the serum. We therefore collected blood from 864 humans ≥13 years of age, 130 bovines and 84 dogs from different rural and urban areas in Bhutan and tested the serum for antibodies specific for leptospires with a screening of enzyme-linked immunosorbent assays (ELISA) and a confirmatory microscopic agglutination test (MAT). In humans, 17.6% were seropositive by ELISA and 1.6% by MAT. The seropositivity was stronger in bovines (36.9%) and dogs (47.6%). “Having had a fever recently” (OR 5.2, *p* = 0.004), “working for the military” (OR 26.6, *p* = 0.028) and “being unemployed” (OR 12.9, *p* = 0.041) (reference category = housemaker) were statistically significantly associated with seropositivity when controlled for the effects of other risk factors. However, due to the small number of positive test results, the findings on risk factors should be interpreted with caution. Based on the serogroups found in the three species, dogs could be a source of infection for humans, or dogs and humans are exposed to the same environmental risk factors Clinical leptospirosis in humans and domestic animals should be investigated by testing blood and urine for the presence of leptospires by molecular methods (qPCR).

## 1. Introduction

Leptospirosis is a neglected worldwide zoonotic bacterial disease and with a high prevalence in subtropical and tropical countries. The estimated global annual incidence is 1.03 million human cases and 58,900 deaths [1]. The taxonomy of leptospires is based on either serological or molecular classification [2]. Serological taxonomy divides *Leptospira* (*L.*) into more than 300 serovars on the basis of surface antigens. Antigenically, related serovars are grouped into serogroups. The molecular classification system groups leptospires on DNA relatedness and is currently at 22 species that are separated into three clusters: “saprophytes”, “intermediates” and “pathogens” [3,4,5,6,7]. However, the description of new species is occurring regularly with the application of whole-genome sequencing [8]. 

Most serovars are adapted to a range of specific wild and domesticated mammals. With a few exceptions, carrier animals exhibit no clinical signs and may transmit leptospires to other animal species and humans via direct contact with urine and aborted tissues, or indirectly through contaminated water and soil [5,9,10]. Leptospirosis among livestock may lead to economic loss for farmers due to abortions, reduced fertility, milk drop or reduced weight gain. Humans may develop a severe or life-threatening illness as accidental hosts following infection, with case fatality rates reaching 15% [5,9,10]. Various antibiotics are effective if started early during the acute stage of illness [2]. In the acute phase, diagnosis is attempted by the detection of specific leptospiral DNA in blood, urine or cerebrospinal fluid using PCR methods. The gold standard, or the serological microscopic agglutination test (MAT), provides tentative information on the infecting serovars but has poor sensitivity during the acute phase. Serological assays like enzyme-linked immunosorbent assays (ELISA) and rapid diagnostic tests share similar disadvantages of low sensitivity in the early disease phase. However, in convalescent sera, seropositivity is much greater [11,12]. 

Neighboring countries of Bhutan, such as India, Nepal and Bangladesh, report leptospirosis as an important contributor to febrile illness with a high seroprevalence in certain regions [13,14,15]. However, the leptospirosis burden in Bhutan is unknown. Leptospirosis has been listed as a notifiable disease in the national Bhutanese “notifiable diseases surveillance manual” since 2008 [16]. The only peer reviewed scientific article we found on leptospirosis in Bhutan (keywords in Web of Science: “Leptospir*” AND “Bhutan”) described a survey in rodents (*n* = 12) in Gedu, a semi-urban town. In one rat, *L. interrogans* was detected by qPCR [17]. In an unpublished report from 2009, seroscreening of cattle at the National Jersey Breeding Centre in the Samtse district indicated the presence of serovar (sv) Hardjobovis with a seroprevalence of 5%. A second unpublished report from a serosurvey in 2014–2015 found 138 of 520 cattle to be positive for sv Lai Like (36.2%), followed by sv Hebdomadis (21.7%) and sv Pomona (15.9%) [18]. In Bhutan, *Leptospira* spp. vaccination is not practiced in any animal species (T. Tshokey, personal communications).

The majority of Bhutan’s population (*n* = 734,374 in 2018 [19]), live in a rural setting and the main occupation is agriculture. Close contact to livestock and dogs occurs on a daily basis and rodents infesting homes are a common sight. Although medical professionals suspect that leptospirosis may be contributing to a substantial number of febrile illness cases in the community, information on the leptospirosis burden and published data are currently lacking. 

The objectives of our research were to identify exposure to *Leptospira* spp. in eight districts of Bhutan by determining the seroprevalence in humans, bovines and dogs. We also aimed to understand whether animals are exposed to and play a role as potential carriers of leptospires. Further objectives were to investigate potential risk factors for seropositivity in humans, such as profession, contact to animals, rural vs. urban lifestyle or altitude, in order to inform the public health sector.

## 2. Methods

### 2.1. Study Populations, Samples and Data Collection 

Human component: We used serum samples and epidemiological data, which were collected in a cross-sectional study from January to March 2015 during the dry winter and early spring season, to establish the seroprevalence of rickettsial antibodies in the healthy Bhutanese population, as described elsewhere [20] (Questionnaire to be found in “Supplementary Material”). In brief, 8 districts from Bhutan’s 20 districts were selected through a probability proportionate to size method (PPS, multistage cluster sampling). The eight districts are listed in Table 1. From each district, a rural and an urban area were selected by the same method resulting in 16 sampling sites in total (8 urban and 8 rural). Each of the 8 selected districts contributed 108 households (76 rural and 32 urban), taken from a list developed during previous national surveys. After the selection of a household, all eligible household members (≥13 years old) were listed, and one member was selected randomly. Participation was voluntary and written consent was obtained. 

Trained laboratory personnel collected 4 mL of blood into BD^®^ blood collection vacutainers (red cap), and through a face-to-face interview demographic data and environmental/animal exposure history were obtained (see Table 2).

Animal component: In a cross-sectional study, bovines (cattle *n* = 120, yak *n* = 10) and dogs (*n* = 84) were blood sampled in the areas (but not necessarily the same households) where the human rickettsial seroprevalence study [20] was carried out as part of the same project between January and April 2015 [21].

Veterinarians and field livestock assistants purposively collected blood from the bovines and dogs of owners who voluntarily participated in the study. We do not know whether several bovines per herd were sampled. However, since the average cattle herd size in Bhutan is four [22], we can expect that many different herds were sampled (in methods, we describe how this problem was statistically treated). Each sampled dog represented one household. Unfortunately, no epidemiological information was collected.

The blood (both animal and human) was immediately taken to the nearest hospital laboratory, and the serum was separated and stored at 2–4 °C until the shipment to the Jigme Dorji Wangchuck National Referral Hospital Laboratory in Thimpu. At this laboratory, all samples were stored at −80 °C until the shipment to the Australian Rickettsial Reference Laboratory (ARRL) in 2015, where they were tested for rickettsial-specific antibodies [20]. 

In 2019, the samples were shipped to the Medical Diagnostic Laboratory of the Swiss Tropical and Public Health Institute (Swiss TPH) to be tested for leptospiral antibodies as described in this article. 

### 2.2. Serological Testing

#### ELISA and Microscopic Agglutination Test

**Figure 1:**
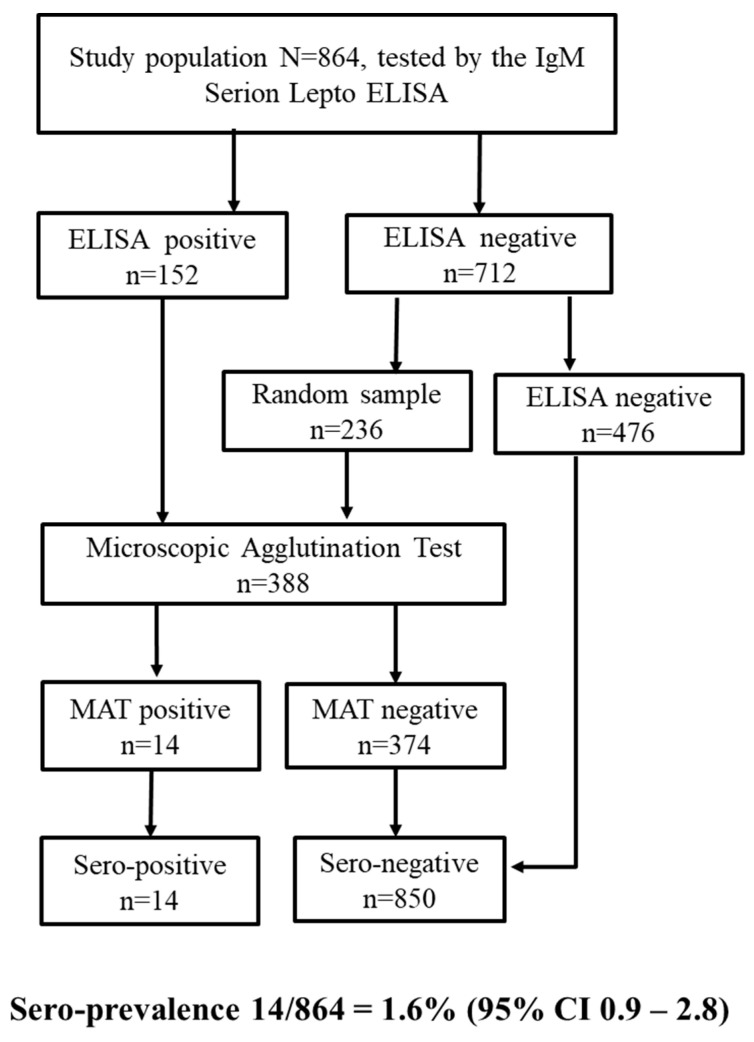
Diagnostic test algorithm used to define *Leptospira* spp. seropositive and negative persons, estimating the prevalence of *Leptospira* spp.-specific antibodies in the human study population in Bhutan.

We screened all 864 human sera with the commercial *Leptospira* IgM Serion ELISA (Serion-Viron, REF ESR125M, referred to in the text as ELISA) according to the manufacturers protocol at the Swiss TPH, and we subsequently sent a subset of these serum samples to the National Consultant Laboratory for *Leptospira* at the German Federal Institute for Risk Assessment (BfR) in Berlin, Germany to be tested by the MAT, including all the ELISA-positive results and a random sample of the ELISA-negative results (Figure 1). Further, we sent all 314 animal sera to the Institute for Infectious Diseases and Zoonoses at Ludwig-Maximilians-University (LMU) in Munich, Germany to be tested by the MAT. 

The presence of antibodies against pathogenic *Leptospira* spp. was assessed with a MAT according to the standards of the World Organisation of Animal Health (OIE) standards [23]. Live cultures of 11 (dogs and bovines) and 17 (humans) *Leptospira* spp. reference strains were used in this study (Table 3). The human sera were screened at a dilution of 1:50 and the animal sera at a dilution of 1:100 (due to cross-reactions between sv Lai and Icterohaemorrhagiae at 1:50). Those with a positive reaction were titrated in a serial two-fold dilution to determine the end-point titer, defined as the reciprocal of the highest serum dilution at which ≥50% of the leptospires remain agglutinated. The same technique was applied at the BfR and the LMU.

### 2.3. Data Analysis

Data was recorded in Microsoft Excel and analyzed with Stata 15. 

#### 2.3.1. Case Definitions

We considered a person or animal positive for *Leptospira* spp.-specific antibodies against any serovar with a MAT titer ≥100 [24], independent of the ELISA serostatus. The human serum samples, which were ELISA-negative and were not tested by MAT, were categorized as seronegative (Figure 1). 

#### 2.3.2. Humans

Proportions of seropositive humans overall, and for each serogroup and serovar listed in Table 1, were calculated. We used the Fleiss Method to calculate 95% confidence intervals for proportions [25]. The distribution of observations and the difference of seroprevalence was described by the following explanatory variables: (1) gender; (2) age; (3) occupation; (4) altitude; (5) whether they live in a rural or urban area (geographical distribution); whether they had contact with (6) sheep, (7) yaks, (8) cattle, (9) dogs, (10) cats or (11) horses; (12) having had a fever in the recent past; or being seropositive against Rickettsia from (13) Spotted Fever Group (SFG), (14) Typhus Group (TG), (15) Scrub Typhus Group (STG) or (16) Q-fever (Table 2). 

Univariable analysis was performed by the Fishers exact test if there were less than five data points in a cell of the 2 × 2 table, and/or by logistic regression analysis to assess the association between *Leptospira* spp. seropositivity and the risk factors (explanatory variables) 1–17 described in the paragraph above. In a manual forward selection method, we then assessed the association between these explanatory variables and *Leptospira* spp. seropositivity by a multivariable logistic regression analysis. Exposure variables were entered in the model if they had a *p*-value *p* ≤ 0.2 in the univariable analysis and were kept in the model if the likelihood ratio test was statistically significant (*p* ≤ 0.05) compared to the nested model. For post-diagnostic statistics, we applied the Pearson χ^2^ statistic to test the goodness-of-fit of the model, and the link test for a linear predicted value (“_hat”) and linear predicted value squared (“_hatsq”).

#### 2.3.3. Bovines and Dogs

Proportions of seropositive bovines and dogs overall and for each serogroup and serovar, as listed in Table 3, were calculated. Seroprevalence data were stratified by district. Cluster robust standard errors were calculated for the 95% confidence intervals to control for potential clustering by herd (bovines). Since we did not know which bovines belonged to the same herd, we assumed that they were clustered by sampling area. 

Human and animal prevalence data were compared to see a potential correlation/clustering by region.

## 3. Results 

### 3.1. Humans 

Study participants were 864 healthy individuals ≥13 years of age. The study population’s characteristics are shown in Table 2, and have been described by Tshokey et al. [20]. Briefly, more females (60.1%) than males (39.9%) participated in the study. Various occupations were well represented with 52.7% of participants being farmers or herders, 15.6% being employees and 21% being homemakers. 

#### 3.1.1. Serological Tests

The ELISA screening resulted in 152 seropositive and 712 seronegative samples. A subset of those samples (*n* = 388) was tested by the MAT at the BfR in Berlin. The 388 samples tested at the BfR, included all the ELISA-positive results (*n =* 152) and a random sample of the ELISA-negative results (*n =* 236). Of the 388 serum samples tested by the MAT, 14 persons showed leptospiral-specific antibodies (Figure 1). 

#### 3.1.2. ELISA Performance Based on the MAT

Of the 14 MAT-positive sera, 12 were ELISA-positive and 2 were ELISA-negative. Of the 374 MAT-negative results, 234 samples were ELISA-negative and 140 were ELISA-positive (Figure 1). If we consider the MAT as the reference test, the ELISA produced 140 false-positive and 2 false-negative test results, leading to a sensitivity of 85.7% (95% CI 57.2%–98.2%) and a specificity of 62.6% (95% CI 57.4%–67.5%). Details of the ELISA performance compared to the MAT are currently being published elsewhere.

#### 3.1.3. Seroprevalence

The prevalence against *Leptospira* spp.-specific antibodies, based on our ELISA screening, was 17.6% (95% CI 15.1–20.3). However, when considering the MAT as the reference test, the *Leptospira* spp. seroprevalence (against any serovar) of the entire study population was lower at 1.6% (95% CI 0.9–2.8, 14/864 persons). It should be noted that we included the ELISA-negative sera, which were not tested by the MAT, in the calculation of the overall seroprevalence (the denominator = total study population; see case definitions). The potential influence of including the ELISA-negative sera on our results is discussed below. The serogroups (sg)/serovars (sv) reacting in the MAT were sg Australis sv Bratislava (*n* = 8), sg/sv Australis (*n* = 3), sg/sv Bataviae (*n* = 2), sg/sv Canicola (*n* = 2), sg/sv Pyrogenes (*n* = 1) and sg/sv Hebdomadis (*n* = 1), with MAT titers ranging from 100 to 200. One cross-reaction occurred between sv Pyrogenes and sv Bratislava, and two between sv Australis and sv Bratislava (same serogroup; data unshown). The *Leptospira* spp. seroprevalence stratified by serogroup and serovar is shown in Table 4, and by the explanatory variables 1–17 in Table 2. Apart from Thimphu and Trongsa, all districts had seropositive persons present (Table 1).

#### 3.1.4. Risk Factors for Seropositivity

The univariable analysis revealed a statistically significant association between the outcome of seropositivity and the risk factors “occupation” (*p* = 0.003) and “had fever recently” (*p* = 0.008). Seropositive cases occurred among all occupational categories with military personnel having the highest percentage (11%, OR 22.6, *p* = 0.03) (Table 2). Persons who recently had a fever were 4.2 times more likely to be seropositive compared to those who did not have a fever recently. Unfortunately, too many data points on animal contacts were missing and could not be further analyzed. All other variables listed in Table 2 were not statistically significantly associated with *Leptospira* spp. serostatus. Given the low number of seropositive cases, we did not look at serogroup specific outcomes, but used the overall *Leptospira* spp. serostatus (including all serovars) as an outcome. The results are shown in Table 2.

In the multivariable logistic regression model, we first entered the variables “had fever recently” and then “occupation” (with the categories “homemaker” (reference category), “herder or farmer”, “employee”, “military”, “student” and “unemployed”) as risk factors for the outcome “*Leptospira* spp. serostatus”. This model remained as the final model, as none of the other variables improved the model fit, based on the likelihood ratio test. The variable “had fever recently” had missing values; hence, the data analysis was reduced to 843 individuals. Study participants who recently had a fever were 5.2 times more likely to have anti-leptospiral antibodies than those who did not have a fever when controlled for the effect of their occupation (*p* = 0.004). Working for the military (OR 26.6, *p* = 0.028) and being unemployed (OR 12.9, *p* = 0.041) remained statistically significantly associated with seropositivity when controlled for the effect of having had a fever recently (Table 5). The Pearson χ^2^ goodness-of-fit test indicated a sufficient fit of the data (*p* = 0.169). However, the variable “_hat” was not a statistically significant predictor (*p* = 0.250).

### 3.2. Animals

#### 3.2.1. *Leptospira* spp. Seroprevalence in Bovines

**Figure 2:**
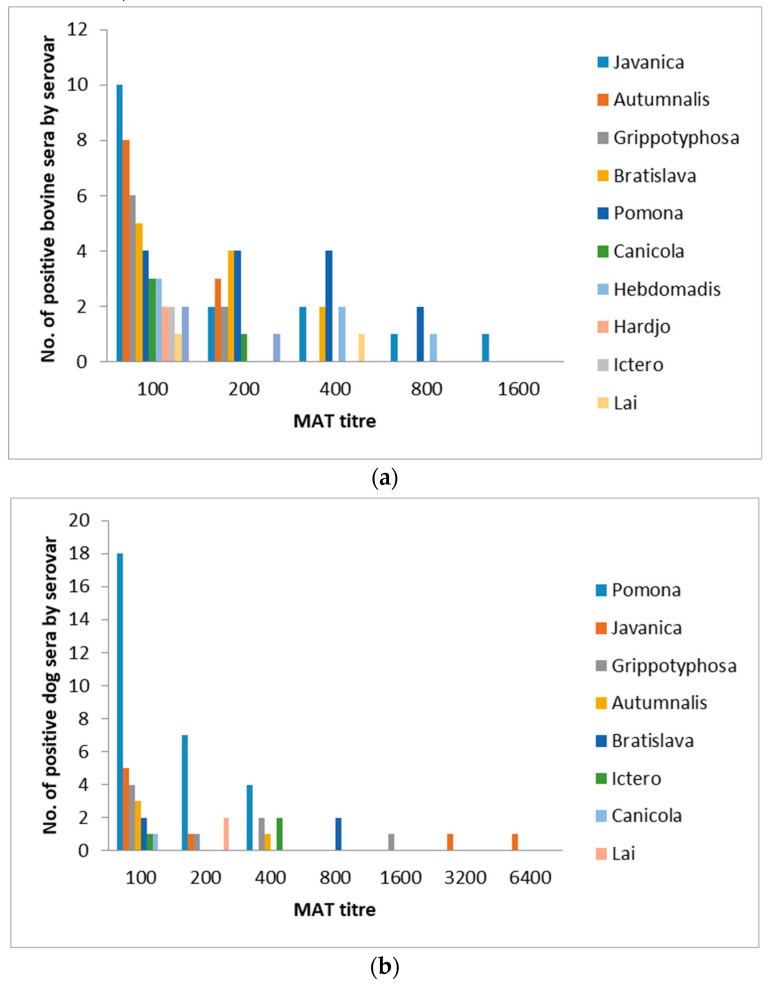
Frequency histogram showing the number of seropositive sera of (**a**) bovines (*n* = 130) and (**b**) dogs (*n* = 84) from several districts in Bhutan at each MAT titer to different *Leptospira* serovars (one animal may be seropositive against several serovars).

The bovine study population was composed of 10 yaks and 120 cattle. The overall *Leptospira* spp. seroprevalence was 36.9% (95% CI 24.1–51.9) with 48 out of 130 bovines being positive against one serovar. The positive MAT titers ranged from 100–1600 (Figure 2). The seroprevalence stratified by serovar is shown in Table 4, and by region/district in Table 1. The highest seroprevalence was tested for sg/sv Javanica (12.3%), followed by sg/sv Pomona (10.8%) and sg Australis/sv Bratislava and sg/sv Autumnalis with 8.5% each (Table 4). The seroprevalence statistically significantly differed by district (*p* = 0.003), with Punakha having the highest proportion of bovines with leptospiral-specific antibodies (71.4%). However, this result came from a very small sample size of seven animals. Further, Zhemgang and Mongar found high prevalences with 65% and 45%, respectively (Table 1).

#### 3.2.2. *Leptospira* spp. Seroprevalence in Dogs

The overall prevalence was 47.6% (95% CI 36.7–58.7), with 40 out of 84 dogs having anti-leptospiral antibodies against one serovar. The positive MAT titers ranged from 100 to 6400 (Figure 2). The seroprevalence stratified by serovar is shown in Table 4, and by region/district in Table 1. Overall, sg/sv Pomona contributed to the seroprevalence with 39.3%, followed by sg/sv Javanica and Grippotyphosa with 9.5% each. The seroprevalence did not differ significantly by district (*p* = 0.647), ranging from 33%–75%.

There were not enough human seropositive cases to analyze an overlap of the clustering of human and animal seropositive cases.

## 4. Discussion

### 4.1. Leptospira spp. Seroprevalence and Risk Factors in Humans and Animals

While few human study participants showed leptospiral-specific antibodies (1.6%, based on the MAT), the bovine and dog populations seemed to have a higher exposure to leptospires, with an overall seroprevalence of 36.9% and 47.6%, respectively. 

Given the random probability proportionate to size, a multistage cluster sampling method applied to collect the human samples, the measured seroprevalence is most likely representative for the Bhutanese population. With 455 study participants coming from the farming/herding sector, the sample size was large enough to estimate a precise *Leptospira* spp. seroprevalence in this stratum. However, only 0.9% of persons from the farming/herding sector were seropositive, despite the rather high *Leptospira* spp. seroprevalence in bovines. Farming or herding was not a risk factor for *Leptospira* spp. seropositivity in the multivariable model. Further, the serogroups detected in humans were not specifically associated with serogroups carried by bovines [2]. The majority of Bhutanese are Buddhist and do not slaughter their own animals. This factor, or other unknown contact patterns, could be one reason for a low infection risk in humans from their animals. Overall, we cautiously interpreted these results to mean that bovines do not play an important role in the transmission of leptospires to the human population in Bhutan.

We detected sg Australis (sv Bratislava) in the human, dog and bovine populations and sg Pomona in the latter two. Hence, it is possible that domestic or wild pigs, which are frequent carriers of Pomona [2,26], may play a role as carriers and in the transmission of leptospires to other animal (including the human) species in Bhutan. Other serovars, such as Icterohemorrhagiae and Grippotyphosa, could be rodent-associated [24].

Based on the prevalent serogroups in humans (Canicola and Australis), dogs may play a role as transmitters of *Leptospira* spp. to humans, or humans and dogs may be exposed to some of the same environmental risk factors. However, similar to bovines, a strong discrepancy between the proportion of seropositive cases in humans and dogs prevails. As with bovines, the low transmission to humans may be due to contact patterns, as dogs are mainly kept outside the houses. However, the capacity of the MAT to detect the actual infecting serogroup is disputed, where some studies demonstrate a poor correlation between the serogroup testing positive on the MAT and the actual infecting serogroup [27,28], and some report a good correlation [29]. Hence, because of cross-reactions in the MAT and changes of the maintenance host status over time due to adaptation patterns [30], the interpretation of transmission pathways based on serogroups must always be done with a lot of caution and should be confirmed by molecular analyses of the isolates. We found two statistically significant risk factors for humans being seropositive against *Leptospira* spp. in the univariable and multivariable analysis: (1) “had a fever recently” and (2) “occupation”, namely the categories “working in the military” and “being unemployed”. Both of these categories are not typical risk factors for *Leptospira* spp. infection. However, they could be a proxy for environmental exposure. Unfortunately, the questionnaire did not include questions on environmental exposure (type of water source used, rice paddy field work, etc.). Given the small number of seropositive cases, we do not want to over interpret these findings. 

What are the findings of leptospirosis studies from countries in proximity to Bhutan? Shrestha et al. [31] collected paired blood samples and data on risk factors among 239 adult, febrile patients in healthcare centers in the mountainous Kaski District of Nepal in 2013. Additionally, animals were blood sampled in 119 patient households: 63 cattle, 92 buffalo, 181 goats and 20 dogs. Serology was performed using the MAT (panel: 20 serovars). In humans, 5.4% (95% CI 2.6–8.3) had clinical leptospirosis (MAT titer ≥1:400 or a ≥4-fold rise between acute and convalescent titers). Owning goats (OR 1.3, CI 95% 1.05–1.66), working in rice fields (OR 1.3, CI 95% 1.11–1.72) and male gender (OR 4, CI 95% 1.12–17.26) significantly increased the risk of clinical leptospirosis in humans. In another fever study among 144 patients in the lowland Terai region of Nepal, 30 (21%) were positive for leptospiral IgM by ELISA [13]. These studies suggest that leptospirosis plays a role in fever patients in the region. It may be an indication that an acute undifferentiated fever (AUF) study would be a useful approach to assess the relevance of leptospirosis in Bhutan. 

In a cross-sectional study from 2019 to 2020, 206 cattle herds were visited and 383 serum samples were collected in the Rupandehi district of Nepal. These were tested against *L.* Hardjo with an ELISA (Prionics Lelystad B.V., The Netherlands), and 3.81% tested positive [32]. As in this study, the *L.* Hardjo antibody prevalence was also low in our study in Bhutan. Studies using the MAT targeting a larger serogroup/serovar panel in bovines are more informative and comparable to our results, such as the one described above by Shrestha et al., where the *Leptospira* spp. seroprevalence in ruminants was 41% in cattle (95% CI 29–53, *n* = 63), 37% in buffalo (95% CI 2–47, *n* = 92) and 17% in goats (95% CI 11–22, *n* = 18). The highest seroprevalence was found in dogs (45%, 95% CI 22–68, *n* = 20) [31]. In another cross-sectional study in street dogs in the Kathmandu valley conducted in 2016, the seroprevalence was lower at 11.4% (*n* = 70). However, an Immunocomb Canine Leptospira Antibody Test Kit was used, and hence, the results are not comparable to the MAT [33].

In summary, leptospirosis is prevalent in neighboring countries with a similar ecological setting and socioeconomic and agricultural system. Based on our findings, we hitherto recommend building a (veterinary) hospital and laboratory-based surveillance system and testing undiagnosed fever cases for leptospirosis in humans and dogs, especially during the rainy season. For acute cases, a direct detection of leptospires in blood and urine by qPCR targeting *LipL32* is a good approach [34,35]. By collecting epidemiological data from human and animal patients, a better understanding of risk factors, areas at risk and risk groups will eventually develop.

In cattle, it is recommended to estimate the economic impact of the high *Leptospira* spp. seroprevalence. This is because many animals had antibodies against serogroups, for which they are accidental hosts (and hence could react with clinical symptoms when infected), such as sg Javanica, Autumnalis and Australis. Hence, if an increase in stillbirth, abortions, calf mortality or milk drop is observed, we recommend the diagnostic investigation of leptospirosis. 

Ideally, the source population would be identified for a targeted preventive approach (trapping of rodents, vaccination of dogs, etc.).

### 4.2. Study Design Limitations

To understand transmission patterns, humans and animals of the same households should have been tested. However, in this study, only animals of the same regions were sampled. Since serology only detects previous exposure but does not discover carrier hosts, even when sampling animals and humans in the same household, seroprevalence studies will not reveal transmission routes in comparison to molecular studies. Our study nevertheless gives insight into the overall *Leptospira* spp. seroprevalences in humans and animals in the same regions, and to our knowledge, it is the first published “One Health” study on leptospirosis in Bhutan.

We calculated cluster robust standard errors for the bovine prevalence estimate to control for potential clustering by herd. Because of the missing information about which individuals may have belonged to the same herd, we used the sampling area as the cluster variable. However, herd size is smaller than the number of bovines found in a sampling area. Hence, we overcontrolled for clustering, probably resulting in wider confidence intervals and a prevalence estimate that is more precise than shown in Table 3. 

The study was conducted during the dry period with the environmental component being of less importance (fewer inundations). This could have contributed to a lower seroprevalence estimate [36]. Nevertheless, antibodies do generally prevail between months and years, so a past exposure from the last rainy season should have still been detectable at a low titer in a proportion of persons and animals.

The serogroup/serovar panel was chosen on the basis of the limited information available on prevalent serogroups in Bhutan [18] and on commonly prevalent serogroups worldwide. However, the panel may have not covered all endemic/local strains with the consequence that the overall prevalence may have been further underestimated. Since testing was targeted towards past exposure to leptospires and not acute disease, a sensitivity of 88% and specificity of 98% can be assumed for the MAT [37]. However, in a Bayesian approach, the estimation of the MAT sensitivity and specificity were even lower, at 54.9% and 97.3% for subclinical cases and 65.6% and 97.7% for clinical cases, albeit in a Central European study population [38]. Therefore, the tested prevalence was most likely underestimated, as the low sensitivity of the MAT can test a proportion of true seropositive cases as false negatives. Using the MAT as the reference test, as was practiced, is problematic because some of the false-positive ELISA test results may have actually been true positives. A poor correlation between an IgM ELISA and the MAT was also reported by Hem et al. [39]; however, the study population constituted acute fever patients. In this case, the authors argued that the ELISA’s higher sensitivity towards IgM antibodies and missing serogroups in the MAT panel led to the discrepancy. Nevertheless, some of our ELISA results were most likely false positives, based on our validation study at the Swiss TPH (publication pending). The reason for those false-positive results is unknown, but as the ELISA plates were coated with an antigen of *L*. biflexa, a non-pathogenic *Leptospira* species which is found ubiquitously in the environment, some unspecific cross-reactions can be expected, as has been shown elsewhere [40]. Moving the test cutoff for a positive ELISA result towards a higher optical density (OD) would have reduced sensitivity and not majorly improved specificity (unpublished results, Swiss TPH). In this case the criteria for a screening test would not be met anymore. IgM antibodies are generally associated with acute disease and are seldom applied in the diagnostics in cross-sectional studies in healthy populations. However, while IgM antibodies do last for several months, IgG antibodies are sometimes not even induced by infection with *Leptospira* spp.

Because of funding issues, we only tested a subsample of our study population by MAT, albeit with sufficient power (*n* = 388). On the basis of previous experience at the medical diagnostic laboratory at the Swiss TPH (unpublished results), we expected the ELISA to produce few false-negative results (high specificity), and hence, we did not test all ELISA-negative results by MAT. Nevertheless, we counted them as true negatives in our seroprevalence estimation (1.6%). If we had only used the 388 samples tested by the MAT to estimate seroprevalence, we would have introduced a selection bias and the results would no longer have been representative of the Bhutanese population.

In the 388 samples, the ELISA produced two false-negative results (based on the MAT). Assuming a similar proportion in the MAT untested 476 ELISA-negative samples, another 2–4 false-negative results are possible. While the prevalence estimate would not have changed dramatically (18/864 = 2.1%), the selection of statistically significant risk factors in the multivariable logistic regression model may have changed because of the low numbers of seropositives in each category. This is another reason not to over interpret the results on risk factors.

The reading of the MAT results can vary between laboratory scientists by approximately one titer cutoff. Since the human and animal samples were tested in different laboratories, a reading discrepancy between the human and animal seroprevalence results cannot be excluded and could have slightly contributed towards the difference of human and animal *Leptospira* spp. seroprevalence (if the human sera MAT results were read with a more stringent MAT titer cutoff at 1:50). 

In conclusion, based on the MAT results, the *Leptospira* spp. seroprevalence in the Bhutanese population is low (1.6%), but may have been underestimated because of the study design and the MAT being the reference test. Cattle and dogs are more exposed to leptospires than humans. Nevertheless, leptospires are present and should be considered in the differential diagnosis of febrile illnesses in humans and domestic animals. While bovines do not seem to be a source of infection for humans based on the results of this study (prevalent serogroups and risk factors), dogs may pose a higher risk of transmitting *Leptospira* spp. Direct or indirect contact to pigs and wildlife may be an important source of infection for bovines and dogs. However, these hypotheses need confirmation by molecular transmission studies. We recommend the investigation of acute disease with symptoms corresponding to leptospirosis in humans and domestic animals and the screening of wildlife based on molecular tests.

## Figures and Tables

**Table 1:** Number (n) and percentage (%) of sampled persons, bovines and dogs, and *Leptospira* spp. seropositive numbers (N Pos), percentage (%) with its lower and upper 95% Confidence Interval (CI)of persons, bovines and dogs, stratified by districts of residence. While there was no statistically significant difference in *Leptospira* seropositivity by district for humans (*p* = 0.235) and dogs (*p* = 0.647), the bovine seroprevalence differed significantly (*p* = 0.003) by district with the Fisher exact test.

	Humans (N = 864)			Bovines (N = 130)			Dogs (N = 84)		
District	n (%)	N Pos (%)	95% CI	n (%)	N Pos (%)	95% CI	n (%)	N Pos (%)	95% CI
Chukha	108 (12.5)	2 (1.8)	0.32–7.19	20 (15.4)	4 (20.0)	6.61–44.27	- ^1^	-	
Mongar	108 (12.5)	1 (0.9)	0.05–5.80	20 (15.4)	9 (45.0)	23.83–67.95	8 (9.5)	6 (75.0)	35.58–95.54
Punakha	108 (12.5)	4 (2.8)	1.19–9.77	7 (5.4)	5 (71.4)	30.26–94.89	9 (10.7)	3 (33.3)	9.04–69.08
Samtse	108 (12.5)	3 (2.8)	0.72–8.50	21 (16.1)	9 (42.9)	22.59–65.56	15 (17.9)	7 (46.7)	22.28–72.58
Thimphu	108 (12.5)	0 (0.0)	0.08–4.28	20 (15.4)	2 (10.0)	1.75–33.13	20 (23.8)	8 (40.0)	19.98–63.59
Trashigang	108 (12.5)	3 (2.8)	0.72–8.50	12 (9.23)	4 (33.3)	11.27–64.56	12 (14.3)	5 (41.7)	16.50–71.40
Trongsa	108 (12.5)	0 (0.0)	0.08–4.28	10 (7.7)	2 (20.0)	3.54–55.78	10 (11.9)	5 (50.0)	20.14–79.86
Zhemgang	108 (12.5)	1 (0.3)	0.05–5.80	20 (15.4)	13 (65.0)	40.95–83.69	10 (11.9)	6 (60.0)	27.37–86.31

^1^ no data available.

**Table 2:** Number and percentage of sampled persons (*n* = 864) and *Leptospira* spp. seropositive numbers and percentage of persons stratified by the explanatory variables listed below.

Explanatory Variable	Category	n (%) ^1^	N Pos (%) ^2^	*p*-Value ^3^
Gender	Female	519 (60.1)	7 (1.35)	0.584
	Male	365 (39.9)	7 (2.03)	
Age groups (years)	13–20	74 (8.6)	0 (0.0)	0.87
	21–35	305 (35.3)	5 (1.6)	
	36–65	406 (47.0)	8 (2.0)	
	66–98	79 (9.1)	1 (1.3)	
**Occupation**	Herder/Farmer	455 (52.7)	4 (0.9)	**0.009**
	Employee	135 (15.6)	5 (3.7)	
	Military	9 (1.0)	1 (11.1)	
	Student	45 (5.2)	1 (2.2)	
	Homemaker	182 (21.0)	1 (0.5)	
	Unemployed	38 (4.4)	2 (5.3)	
Altitude (m)	High >2000	140 (16.2)	0 (0.0)	0.229
	Middle 1000–2000	584 (67.6)	11 (1.9)	
	Low < 1000	140 (16.2)	3 (2.1)	
Geographical distribution	Rural	608 (70.4)	12 (2.0)	0.252
	Urban	256 (29.6)	2 (0.8)	
Contact to sheep ^4^	No	- ^5^	-	-
	Yes	16	-	-
Contact to yaks	No	-	-	-
	Yes	57	-	-
Contact to cattle	No	-	-	-
	Yes	268	-	-
Contact to dogs	No	-	-	-
	Yes	292	-	-
Contact to cats	No	-	-	-
	Yes	284	-	-
Contact to horses	No	-	-	-
	Yes	74	-	-
**Had fever recently**	No	637 (75.6)	6 (0.9)	**0.008**
	Yes	206 (24.4)	8 (3.9)	
Seropositive to SFG	No	673 (77.9)	12 (1.8)	0.746
	Yes	191 (22.1)	2 (1.0)	
Seropositive to TG	No	834 (96.5)	12 (1.4)	0.082
	Yes	30 (3.5)	2 (6.7)	
Seropositive to STG	No	668 (77.3)	8 (1.2)	0.1
	Yes	196 (22.7)	6 (3.1)	
Seropositive to Q-fever	No	801 (93.0)	13 (1.6)	1.0
	Yes	60 (7.0)	1 (1.7)	

^1^ n (%) is the number and percentage of observations under each variable category; ^2^ N Pos (%) are the number and percentage of Leptospira spp. seropositive persons by variable category; ^3^ Fischer exact test; ^4^ data analysis of contact to animals was not possible, as data was missing and only available for one category; ^5^ no data available. In **bold**: statistically significantly different by category (*p*-value ≤ 0.05). Abbreviations: SFG = Spotted Fever Group, TG = Typhus Group, STG = Scrub Typhus Group.

**Table 3:** Strains of *Leptospira* spp. used as live antigens in the microscopic agglutination test (MAT).

Genomspecies	Serogroup	Serovar	Strain
*L. interrogans*	Australis	Australis	Ballico ^h^
*L. interrogans*	Australis	Bratislava	Jez Bratislava ^bhc^
*L. interrogans*	Autumnalis	Autumnalis	Akiyami A ^bhc^
*L. interrogan* *s*	Bataviae	Bataviae	Swart ^h^
*L. interrogans*	Canicola	Canicola	Hond Utrecht IV ^bhc^
*L. interrogans*	Hebdomadis	Hebdomadis	Hebdomadis ^bhc^
*L. interrogan* *s*	Icterohaemorrhagiae	Copenhageni	M20 ^h^
*L. interrogan* *s*	Icterohaemorrhagiae	Icterohaemorrhagiae	Ictero I ^bc^
*L. interrogans*	Icterohaemorrhagiae	Icterohaemorrhagiae	RGA ^h^
*L. interrogan* *s*	Icterohaemorrhagiae	Lai	Lai ^bc^
*L. interrogans*	Pomona	Pomona	Pomona ^bhc^
*L. interrogans*	Pyrogenes	Pyrogenes	Salinem ^h^
*L. interrogans*	Sejroe	Hardjo	Hardjoprajitno ^bhc^
*L. borgpetersenii*	Ballum	Ballum	Mus 127 ^h^
*L. borgpetersenii*	Javanica	Javanica	Veldrat Batavia 46 ^bhc^
*L. borgpetersenii*	Sejroe	Saxkoebing	Mus 24 ^h^
*L. borgpetersenii*	Sejroe	Sejroe	M 84 ^bhc^
*L. borgpetersenii*	Tarassovi	Tarassovi	Perepelitsin ^h^
*L. kirschneri*	Grippotyphosa	Grippotyphosa	Moskva V ^bhc^

Tested in bovines ^b^, and/or humans ^h^ and/or dogs ^c^.

**Table 4:** Seroprevalence of *Leptospira* serovars (titer ≥100) tested by the MAT among humans (*n* = 864), bovines (*n* = 130) and dogs (*n* = 84) sampled in the same eight districts in Bhutan.

	Humans			Bovines			Dogs		
Serovar	n Pos ^1^	Prev ^2^ %	95% CI ^3^	n Pos ^1^	Prev ^2^ %	95% CI ^4^	n Pos ^1^	Prev ^2^ %	95% CI ^3^
Autumnalis	0	0.00	0.01–0.55	11	8.46	3.34–19.80	4	4.76	1.54–12.40
Bratislava	8	0.93	0.43–1.89	11	8.46	3.49–19.07	4	4.76	1.54–12.40
Canicola	2	0.23	0.04–0.93	4	3.08	0.71–12.32	1	1.19	0.06–7.37
Grippotyphosa	0	0.00	0.01–0.55	8	6.15	2.63–13.75	8	9.52	4.48–18.40
Hardjo	0	0.00	0.01–0.55	2	1.54	0.35–6.41	0	0.00	0.10–5.45
Hebdomadis	1	0.12	0.01–0.75	6	4.62	1.04–18.20	0	0.00	0.10–5.45
Icterohaemorrhagiae ^5^	0	0.00	0.01–0.55	2	1.54	0.18–12.00	3	3.57	0.92–10.80
Javanica	0	0.00	0.01–0.55	16	12.31	6.64–21.70	8	9.52	4.48–18.40
Pomona	0	0.00	0.01–0.55	14	10.77	5.42–20.25	33	39.29	28.99–50.57
Sejroe	0	0.00	0.01–0.55	3	2.31	0.26–17.61	0	0.000	0.10–5.45
Lai	- ^6^	-	-	2	1.54	0.17–12.80	1	1.19	0.06–7.37
Australis	3	0.35	0.09–1.10	-	-	-	-	-	-
Ballum	0	0.00	0.01–0.55	-	-	-	-	-	-
Bataviae	2	0.23	0.04–0.93	-	-	-	-	-	-
Copenhageni	0	0.00	0.01–0.55	-	-	-	-	-	-
Pyrogenes	1	0.12	0.01–0.75	-	-	-	-	-	-
Saxkoebing	0	0.00	0.01–0.55	-	-	-	-	-	-
Tarassovi	0	0.00	0.01–0.55	-	-	-	-	-	-

^1^ Number of seropositives; ^2^ Seroprevalence; ^3^ Fleiss-Method was used to calculate 95% confidence intervals (CI); ^4^ cluster robust standard errors were used to calculate 95% CI, to control for potential clustering; ^5^ two different strains were used for humans (RGA, see Table 3) and bovines/dogs (Ictero I, see Table 3); ^6^ no data available. The overall seroprevalence (against any serovar) in humans was 1.6% (95% CI 0.9–2.8), with 14 out of 864 persons being seropositive against one serovar. In cattle the overall seroprevalence was 36.9% (95% CI 24.1–51.9) with 48 out of 130 bovines being positive against one serovar, in dogs the overall prevalence was 47.6% (95% CI 36.7–58.7) with 40 out of 84 dogs having anti-leptospiral antibodies against one serovar.

**Table 5:** Multivariable logistic regression model showing the association between the risk factors (explanatory variables) “recently had a fever” and “occupation” and the outcome “*Leptospira* seropositivity” (any serovar) among 843 healthy Bhutanese study participants.

Explanatory Variable	Odds Ratio (OR)	*p*-Value	95% Confidence Interval
Fever	**5.24**	**0.004**	1.70–16.16
Occupation			
Homemaker	reference		
Herder/farmer	1.18	0.886	0.13–10.78
Employee	5.70	0.116	0.65–50.09
Military	**26.65**	**0.028**	**1.44–494.28**
Student	3.41	0.393	0.20–56.83
Unemployed	**12.94**	**0.041**	**1.11–150.94**

In **bold**: statistically significant risk factors (*p*-value ≤ 0.05).

## Data Availability

The data presented in this study are available on request from the corresponding author. The data are not publicly available due to the ethics committee requirements.

## Supplementary Materials

The following are available online at https://www.mdpi.com/2076-0817/10/3/308/s1, File S1: Questionnaire.

## References

[1] Costa F., Hagan J.E., Calcagno J., Kane M., Torgerson P., Martinez-Silveira M.S., Stein C., Abela-Ridder B., Ko A.I. (2015). Global Morbidity and Mortality of Leptospirosis: A Systematic Review. PLoS Negl. Trop. Dis..

[2] Bharti A.R., Nally J.E., Ricaldi J.N., Matthias M.A., Diaz M.M., Lovett M.A., Levett P.N., Gilman R.H., Willig M.R., Gotuzzo E. (2003). Leptospirosis: A zoonotic disease of global importance. Lancet Infect. Dis..

[3] Yasuda P.H., Steigerwalt A.G., Sulzer K.R., Kaufmann A.F., Rogers F., Brenner D.J. (1987). Deoxyribonucleic-Acid Relatedness between Serogroups and Serovars in the Family Leptospiraceae with Proposals for 7 New Leptospira Species. Int. J. Syst. Bacteriol..

[4] Ramadass P., Jarvis B.D.W., Corner R.J., Penny D., Marshall R.B. (1992). Genetic-Characterization of Pathogenic Leptospira Species by DNA Hybridization. Int. J. Syst. Bacteriol..

[5] Hartskeerl R.A., Collares-Pereira M., Ellis W.A. (2011). Emergence, control and re-emerging leptospirosis: Dynamics of infection in the changing world. Clin. Microbiol. Infect..

[6] Cerqueira G.M., Picardeau M. (2009). A century of Leptospira strain typing. Infect. Genet. Evol..

[7] Thibeaux R., Iraola G., Ferrés I., Bierque E., Girault D., Soupé-Gilbert M.-E., Picardeau M., Goarant C. (2018). Deciphering the unexplored Leptospira diversity from soils uncovers genomic evolution to virulence. Microb Genom..

[8] Caimi K., Ruybal P. (2020). Leptospira spp., a genus in the stage of diversity and genomic data expansion. Infect. Genet. Evol. J. Mol. Epidemiol. Evol. Genet. Infect. Dis..

[9] Faine S., Adler B., Bolin C., Perolat P. (1999). Leptospira and Leptospirosis.

[10] Adler B., de la Pena Moctezuma A. (2010). Leptospira and leptospirosis. Vet. Microbiol..

[11] Lubell Y., Althaus T., Blacksell S.D., Paris D.H., Mayxay M., Pan-Ngum W., White L.J., Day N.P., Newton P.N. (2016). Modelling the Impact and Cost-Effectiveness of Biomarker Tests as Compared with Pathogen-Specific Diagnostics in the Management of Undifferentiated Fever in Remote Tropical Settings. PLoS ONE.

[12] Lim C., Paris D.H., Blacksell S.D., Laongnualpanich A., Kantipong P., Chierakul W., Wuthiekanun V., Day N.P., Cooper B.S., Limmathurotsakul D. (2015). How to Determine the Accuracy of an Alternative Diagnostic Test when It Is Actually Better than the Reference Tests: A Re-Evaluation of Diagnostic Tests for Scrub Typhus Using Bayesian LCMs. PLoS ONE.

[13] Regmi L., Pandey K., Malla M., Khanal S., Pandey B.D. (2017). Sero-epidemiology study of leptospirosis in febrile patients from Terai region of Nepal. BioMed Cent. Infect. Dis..

[14] Victoriano A.F.B., Smythe L.D., Gloriani-Barzaga N., Cavinta L.L., Kasai T., Limpakarnjanarat K., Ong B.L., Gongal G., Hall J., Coulombe C.A. (2009). Leptospirosis in the Asia Pacific region. BioMed Cent. Ltd Infect. Dis..

[15] Bhattachan B., Jb S., Bg D., Sherchand J. (2016). Leptospirosis: An Emerging Infectious Disease in Nepal. J. Inst. Med..

[16] (2008). Public Health Laboratory Ministry of Health, Notifiable Disease Surveillance Manual. Department of Public Health. http://www.rcdc.gov.bt/web/wp-content/uploads/2015/07/Notifiable-Disease-Surveillance-Manual.pdf.

[17] Phuentshok Y., Dorji K., Zangpo T., Davidson S.A., Takhampunya R., Tenzinla T., Dorjee C., Morris R.S., Jolly P.D., Dorjee S. (2018). Survey and Phylogenetic Analysis of Rodents and Important Roden Borne Zoonotic Pathogens in Gedu, Bhutan. Korean J. Parasitol..

[18] Tenzin T. (2015). Risk Based Surveillance of Leptospirosis in Cross-Species Domestic Animals in Bhutan.

[19] National Statistics. Bureau Bhutan at a Glance 2019. http://www.nsb.gov.bt/publication/files/pub1ai6514dg.pdf.

[20] Tshokey T., Stenos J., Durrheim D.N., Eastwood K., Nguyen C., Graves S.R. (2017). Seroprevalence of rickettsial infections and Q fever in Bhutan. PLoS Negl. Trop. Dis..

[21] Tshokey T., Stenos J., Tenzin T., Drukpa K., Gurung R.B., Graves S.R. (2019). Serological Evidence of Rickettsia, Orientia, and Coxiella in Domestic Animals from Bhutan: Preliminary Findings. Vector Borne Zoonotic Dis..

[22] Wangdi J., Mindu, Bhujel P., Karma, Wangchuk S. (2014). Productive and reproductive performance of dairy cattle and their crossbreds in Bhutan. Livest. Res. Rural. Dev..

[23] (2018). Office International des Epizooties (OIE), Leptospirosis. OIE Terrestrial Manual.

[24] Levett P.N. (2001). Leptospirosis. Clin. Microbiol. Rev..

[25] Fleiss J.L. (1981). Statistical methods for RATES and Proportions.

[26] Jansen A., Luge E., Guerra B., Wittschen P., Gruber A.D., Loddenkemper C., Schneider T., Lierz M., Ehlert D., Appel B. (2007). Leptospirosis in urban wild boars, Berlin, Germany. Emerg. Infect. Dis.

[27] Levett P.N. (2003). Usefulness of Serologic Analysis as a Predictor of the Infecting Serovar in Patients with Severe Leptospirosis. Clin. Infect. Dis..

[28] Chirathaworn C., Inwattana R., Poovorawan Y., Suwancharoen D. (2014). Interpretation of microscopic agglutination test for leptospirosis diagnosis and seroprevalence. Asian Pac. J. Trop. Biomed..

[29] Blanco R.M., dos Santos L.F., Galloway R.L., Romero E.C. (2016). Is the microagglutination test (MAT) good for predicting the infecting serogroup for leptospirosis in Brazil?. Comp. Immunol. Microbiol. Infect. Dis..

[30] Dreyfus A., Wilson P., Benschop J., Collins-Emerson J., Verdugo C., Heuer C. (2018). Seroprevalence and herd-level risk factors for seroprevalence of Leptospira spp. in sheep, beef cattle and deer in New Zealand. N. Z. Vet. J..

[31] Shrestha R., McKenzie J.S., Gautam M., Adhikary R., Pandey K., Koirala P., Bc G.B., Miller L.C., Collins-Emerson J., Craig S.B. (2018). Determinants of clinical leptospirosis in Nepal. Zoonoses Public Health.

[32] Gompo T.R., Jyoti S., Pandit S., Sapkota R.C., Pandey A. (2020). Sero-prevalence and risk factors of leptospirosis in commercial cattle herds of Rupandehi district, Nepal. bioRxiv.

[33] Ojha K.C., Singh D.K., Kaphle K., Shah Y., Pant D.K. (2018). Sero-prevalence of leptospirosis and differentiation in blood parameters between positive and negative cases in dogs of Kathmandu Valley. Trans. R. Soc. Trop. Med. Hyg..

[34] Alinaitwe L., Kankya C., Allan K.J., Rodriguez-Campos S., Torgerson P., Dreyfus A. (2019). Bovine leptospirosis in abattoirs in Uganda: Molecular detection and risk of exposure among workers. Zoonoses Public Health.

[35] Goarant C. (2016). Leptospirosis: Risk factors and management challenges in developing countries. Res. Rep. Trop. Med..

[36] Khanal D., Paudyal N., Khanal S., Prajapati M., Shrestha M., Bowen R., Acharya M., Shrestha S., Singh U., Thakur R. (2018). Detection of Antibodies Against Leptospira hardjo in Large Ruminants of Nepal. Acta Sci. Agric..

[37] Goris M. (2015). Diagnostic tests for human leptospirosis. Second Meeting of the European Leptospirosis Society on Leptospirosis and Other Rodent Borne Hemorrhagic Fevers.

[38] Schlichting D., Nöckler K., Bahn P., Luge E., Greiner M., Müller-Graf C., Mayer-Scholl A. (2015). Estimation of the sensitivity and specificity of a Leptospira spp. in-house ELISA through Bayesian modelling. Int. J. Med. Microbiol..

[39] Hem S., Ly S., Votsi I., Vogt F., Asgari N., Buchy P., Heng S., Picardeau M., Sok T., Ly S. (2016). Estimating the Burden of Leptospirosis among Febrile Subjects Aged below 20 Years in Kampong Cham Communities, Cambodia, 2007-2009. PLoS ONE.

[40] Niloofa R., Fernando N., De Silva N.L., Karunanayake L., Wickramasinghe H., Dikmadugoda N., Premawansa G., Wickramasinghe R., De Silva H.J., Premawansa S. (2015). Diagnosis of Leptospirosis: Comparison between Microscopic Agglutination Test, IgM-ELISA and IgM Rapid Immunochromatography Test. PLoS ONE.

